# Optimal sequencing of chemotherapy with chemoradiotherapy based on TNM stage classification and EBV DNA in locoregionally advanced nasopharyngeal carcinoma

**DOI:** 10.1186/s40880-019-0398-0

**Published:** 2019-10-25

**Authors:** Li-Ting Liu, Melvin L. K. Chua, Yungan Tao, Lin-Quan Tang, Hai-Qiang Mai

**Affiliations:** 1 Sun Yat-sen University Cancer Center; State Key Laboratory of Oncology in South China; Collaborative Innovation Center for Cancer Medicine; Key Laboratory of Nasopharyngeal Carcinoma Diagnosis and Therapy, Guangzhou, 510060 Guangdong P. R. China; 2 Department of Nasopharyngeal Carcinoma, Sun Yat-sen University Cancer Center, 651 Dongfeng Road East, Guangzhou, 510060 Guangdong P. R. China; 3 Divisions of Radiation Oncology and Medical Sciences, National Cancer Centre Singapore, Singapore, 169610 Singapore; 4 Oncology Academic Programme, Duke-National University of Singapore Medical School, Singapore, 169857 Singapore; 5 Institute Gustave-Roussy, 94800 Villejuif, France

## Main text

In the past decades, there have been several studies concerning the efficacy of sequencing of chemotherapy on disease control and survival in locoregionally advanced (LA) nasopharyngeal carcinoma (NPC). The addition of concurrent cisplatin to radiotherapy has demonstrated survival improvements that are attributable to both distant metastasis and locoregional control. Specific to the latter, the advent of intensity-modulated radiotherapy has resulted in superior tumor control given the better dosimetry compared to conventional techniques [1]. However, distant recurrence still occurs in 20–30% patients and accounts for the main cause of death. To address this, several groups have explored the advantages of adding neoadjuvant chemotherapy (NACT) or adjuvant chemotherapy (ACT) to the backbone of concurrent chemoradiotherapy (CCRT).

Still, controversy remains regarding the superiority of the NACT or ACT approaches [2–4]. A possible reason for these controversial results could be the therapeutic decisions in the aforementioned studies were based primarily on the TNM stage for risk stratification. However, it is known that patients with similar stages have markedly different prognoses, and thus additional complementary prognostic and predictive biomarkers are needed in NPC. In endemic regions where the majority of NPC cases are associated with Epstein-Barr virus (EBV) infection, circulating cell-free EBV DNA that is being released by both the replicating and dead tumor cells can be a quantifiable biomarker to complement the TNM stage classification [5]. Of note, plasma EBV DNA load at baseline and post-treatment has been used for predicting survival outcomes of NPC patients [6]. In this regard, two randomized clinical trials were designed to investigate the role of using post-treatment EBV DNA to personalize treatment intensity in the adjuvant setting (NRG-HN001 [NCT02135042] and NPC-0502 [7]).

Recently, a study published in the *Journal of the National Comprehensive Cancer Network* entitled “Neoadjuvant or Adjuvant Chemotherapy Plus CCRT Versus CCRT Alone in the Treatment of Nasopharyngeal Carcinoma: A Study Based on EBV DNA” explored the value of adding NACT or ACT to CCRT in locoregionally advanced NPC patients, who have been stratified into disparate risk groups of distant metastasis [8]. In this study, patients with stage III-IVb (7th edition of UICC/AJCC stage classification system) disease were classified into three risk groups according to their N-status (N0–1 vs. N2–3) and baseline plasma EBV DNA before treatment (< 4000 and ≥ 4000 copies/mL). One of our previous studies reported that these factors were significantly correlated with distant metastasis [6]. Briefly, the low-risk group comprised of patients with N0–1 and EBV DNA < 4000 copies/mL; intermediate-risk group consisted of patients with N0–1 status and high EBV DNA (≥ 4000 copies/mL) or N2–3 status and low EBV DNA (< 4000 copies/mL); and high-risk patients comprised of patients with both adverse risk factors, N2–3 and high EBV DNA (≥ 4000 copies/mL). Based on this risk stratification, we were able to select patients with disparate risk of distant metastasis, for which 6.6%, 14.4% and 26.0% of the patients developed distant metastasis in the low-, intermediate-, and high-risk subgroups, respectively. The efficacy of NACT or ACT to CCRT was then investigated in the different risk groups.

We observed that among the different risk-groups, NACT followed by CCRT significantly reduced the risk of distant metastasis recurrence in the low-risk group (5-year distant metastasis-free survival, 96.2% [NACT + CCRT] vs. 91.3% [CCRT]), but the effect was less apparent in the intermediate-risk group (85.8% vs. 87.3%, respectively). Of note, the NACT regimes that were used included the doublet combinations of taxotere or 5-fluorouracil and cisplatin or the triplet combination of these three drugs. NACT was also more efficacious than CCRT in the high-risk group, albeit this did not reach statistical significance (75.2% [NACT + CCRT] vs. 70.2% [CCRT]). These findings are consistent with a randomized controlled phase 3 trial conducted by Sun et al. which showed that the addition of the triplet combination of taxotere, 5-fluorouracil and cisplatin (TPF) to CCRT improved survival. Interestingly, the investigators found that NACT followed by CCRT offered better distant metastasis-free survival than CCRT alone in the N1-subgroup, but not in the N2–3-subgroups, although admittedly, this was an unplanned subgroup analysis [7]. Based on these observations, we hypothesized that perhaps the strategy of NACT + CCRT would be most efficacious in patients with a low burden of occult metastases, and in patients with a high burden of occult metastases alternative strategies of systemic intensification are needed. It is, however, important to highlight a key confounder here that is the number of cycles of NACT was not controlled in this analysis, and thus we cannot exclude the effect of physician bias on these results (tailoring the intensity of NACT depending on the longitudinal tumor response). Going forward, it is also important to improve the tolerability of NACT in these patients, given that triplet TPF is myelotoxic [9]. More recently, it was shown that the doublet combination of gemcitabine and cisplatin also improves survival when added to CCRT, and this regime seemed better tolerated than TPF [10].

Another interesting observation was that CCRT + ACT yielded the most impressive distant metastasis-free survival than CCRT alone in the high-risk group (5-year: 82.4% vs. 70.2%). This result was in line with findings of the network meta-analysis demonstrated by Ribassin-Majed et al. [11]. However, it is important to point out that this cohort comprised of a very small subgroup of patients (*n* = 53), and therefore over-interpretation of this finding should be avoided. Moreover, 37 (69.8%) of the 53 patients received only 1–2 cycles of ACT and had dose reductions, and hence the improvement in survival was unexpected considering the suboptimal treatment intensity. The role of ACT remains to be defined, and we await the ongoing trials of adjuvant capecitabine (NCT02143388; NCT02958111) and immunotherapy [12], which may shed additional insights on the drug of choice and optimal dosing (metronomic [13] vs. conventional dosing).

Currently, most clinical trials concerning NACT followed by CCRT were primarily conducted in patients at high-risk of treatment failure. The results of this study provided another direction for trials investigating the addition of NACT to CCRT in the low-risk group. Meanwhile, for patients in the intermediate and high-risk group, prescribing combinations of new drugs with reduced toxicities or immune checkpoint inhibitors to improve compliance with ACT or modify the cycles of NACT (more vs. less) might result in improvements in survival. Additionally, these trials will help to validate this method of risk stratifying patients that integrates EBV DNA and TNM-stage classification. Future trials can also be designed to compare the efficacy of NACT plus CCRT versus CCRT followed by ACT, particularly in the high-risk group. Meanwhile, the data presented in the study could help guide clinical practice.

In summary, we provided some insights on the optimal treatment intensity of different risk-groups for locoregionally advanced NPC patients. This is aligned with the direction of future management to personalize the treatment intensity for these patients by employing and monitoring robust biomarkers such as EBV DNA at baseline, post-NACT [14] and prior to ACT. At present, EBV DNA remains the most effective clinically used biomarker, but emerging data from molecular profiling studies will uncover other novel biomarkers that could predict tumor aggression and drug response [15, 16]. Until then, the management of locoregionally advanced NPC patient must entail a detailed discussion with the patient about the potential benefits and risks of toxicities and the impact to quality of life with the different treatment strategies.

## Data Availability

Not applicable.

## References

[1] Peng H, Chen L, Guo R, Zhang Y, Li WF, Mao YP (2017). Clinical treatment considerations in the intensity-modulated radiotherapy era for patients with N0-category nasopharyngeal carcinoma and enlarged neck lymph nodes. Chin J Cancer..

[2] Lee AW, Ngan RK, Tung SY, Cheng A, Kwong DL, Lu TX (2015). Preliminary results of trial NPC-0501 evaluating the therapeutic gain by changing from concurrent-adjuvant to induction-concurrent chemoradiotherapy, changing from fluorouracil to capecitabine, and changing from conventional to accelerated radiotherapy fractionation in patients with locoregionally advanced nasopharyngeal carcinoma. Cancer.

[3] Liu GY, Lv X, Wu YS, Mao MJ, Ye YF, Yu YH (2018). Effect of induction chemotherapy with cisplatin, fluorouracil, with or without taxane on locoregionally advanced nasopharyngeal carcinoma: a retrospective, propensity score-matched analysis. Cancer Commun.

[4] Hui EP, Ma BB, Leung SF, King AD, Mo F, Kam MK (2009). Randomized phase II trial of concurrent cisplatin-radiotherapy with or without neoadjuvant docetaxel and cisplatin in advanced nasopharyngeal carcinoma. J Clin Oncol.

[5] Yao JJ, Zhou GQ, Wang YQ, Wang SY, Zhang WJ, Jin YN (2017). Prognostic values of the integrated model incorporating the volume of metastatic regional cervical lymph node and pretreatment serum Epstein–Barr virus DNA copy number in predicting distant metastasis in patients with N1 nasopharyngeal carcinoma. Chin J Cancer..

[6] Chen YP, Chan ATC, Le QT, Blanchard P, Sun Y, Ma J (2019). Nasopharyngeal carcinoma. Lancet.

[7] Chan ATC, Hui EP, Ngan RKC, Tung SY, Cheng ACK, Ng WT (2018). Analysis of plasma Epstein–Barr virus DNA in nasopharyngeal cancer after chemoradiation to identify high-risk patients for adjuvant chemotherapy: a randomized controlled trial. J Clin Oncol..

[8] Liu LT, Chen QY, Tang LQ, Guo SS, Guo L, Mo HY (2019). Neoadjuvant or adjuvant chemotherapy plus concurrent CRT versus concurrent CRT alone in the treatment of nasopharyngeal carcinoma: a study based on EBV DNA. J Natl Compr Cancer Netw.

[9] Sun Y, Li WF, Chen NY, Zhang N, Hu GQ, Xie FY (2016). Induction chemotherapy plus concurrent chemoradiotherapy versus concurrent chemoradiotherapy alone in locoregionally advanced nasopharyngeal carcinoma: a phase 3, multicentre, randomised controlled trial. Lancet Oncol.

[10] Zhang Y, Chen L, Hu GQ, Zhang N, Zhu XD, Yang KY (2019). Gemcitabine and cisplatin induction chemotherapy in nasopharyngeal carcinoma. N Engl J Med..

[11] Ribassin-Majed L, Marguet S, Lee AWM, Ng WT, Ma J, Chan ATC (2017). What is the best treatment of locally advanced nasopharyngeal carcinoma? An individual patient data network meta-analysis. J Clin Oncol.

[12] Wang YQ, Chen YP, Zhang Y, Jiang W, Liu N, Yun JP (2018). Prognostic significance of tumor-infiltrating lymphocytes in nondisseminated nasopharyngeal carcinoma: a large-scale cohort study. Int J Cancer.

[13] Brandi G, Venturi M, De Lorenzo S, Garuti F, Frega G, Palloni A (2018). Sustained complete response of advanced hepatocellular carcinoma with metronomic capecitabine: a report of three cases. Cancer Commun.

[14] Chua MLK (2019). Circulating tumor DNA to personalize treatment in nasopharynx cancer—time to look “ahead”?. Int J Radiat Oncol Biol Phys.

[15] Li YY, Chung GT, Lui VW, To KF, Ma BB, Chow C (2017). Exome and genome sequencing of nasopharynx cancer identifies NF-kappaB pathway activating mutations. Nat Commun..

[16] Zheng H, Dai W, Cheung AK, Ko JM, Kan R, Wong BW (2016). Whole-exome sequencing identifies multiple loss-of-function mutations of NF-kappaB pathway regulators in nasopharyngeal carcinoma. Proc Natl Acad Sci USA.

